# Vitamin D treatment distinctly modulates cytokine production by peripheral blood mononuclear cells among patients with chronic cardiac and indeterminate clinical forms of Chagas disease

**DOI:** 10.1002/iid3.1330

**Published:** 2024-09-13

**Authors:** Kamila Kássia dos Santos Oliveira, Diego José Lira Torres, Michelle da Silva Barros, Leyllane Rafael Moreira, Claudeir Dias da Silva Junior, Ana Karine de Araújo Soares, Maria da Piedade Costa Reis de Albuquerque, Maria da Glória Aureliano Melo Cavalcante, Wilson Alves de Oliveira Junior, Michelle Christiane da Silva Rabello, Virginia Maria Barros de Lorena

**Affiliations:** ^1^ Institute Aggeu Magalhães, Laboratory of Immunoparasitology Oswaldo Cruz Foundation‐FIOCRUZ Recife Pernambuco Brazil; ^2^ Tropical Medicine Department Federal University of Pernambuco (UFPE) Recife Pernambuco Brazil; ^3^ Altino Ventura Foundation (FAV) Recife Pernambuco Brazil; ^4^ Chagas disease and Heart Failure Outpatient Clinic of the Pronto Socorro Cardiológico de Pernambuco University of Pernambuco (UPE) Recife Pernambuco Brazil

**Keywords:** Chagas disease, immune response, vitamin D

## Abstract

**Introduction:**

Chagas disease is caused by the protozoan *Trypanosoma cruzi* and is clinically divided into acute and chronic phases. Chronic Chagas cardiomyopathy is the most studied manifestation of the disease. Vitamin D deficiency has been suggested as a risk factor for cardiovascular disease. No studies demonstrate the action of this hormone in the cells of patients with chronic Chagas heart disease.

**Objective:**

To evaluate the in vitro immunomodulatory effect of vitamin D on peripheral blood mononuclear cells of patients with the different chronic clinical forms of Chagas disease. Evaluating vitamin D's in vitro effect on blood cells by producing cytokines.

**Methods:**

Thirteen patients of the undetermined form (IND), 13 of the mild cardiac form (CARD1) and 14 of the severe cardiac form (CARD2) of Chagas disease, and 12 with idiopathic heart disease (CARDid) were included. The cells obtained from peripheral blood were treated in vitro with vitamin D (1 × 10^−7 ^M) for 24 h and cytokines were dosed in the culture supernatant.

**Results:**

Although it was not possible to demonstrate statistically significant differences between the groups studied, our data showed that the cells treated with vitamin D modify (*p* < .05) the production of interferon‐γ (IFN‐γ) (decrease in IND), tumor necrosis factor‐α (TNF‐α) (decreased in CARD1 and CARDid), interleukin (IL)‐6 (increased in all groups), and IL‐10 (decreased in CARD1, CARD2, and CARDid) when compared to untreated cells.

**Conclusion:**

In vitro treatment with vitamin D distinctly modulated the production of cytokines by mononuclear cells of peripheral blood among patients with chronic and indeterminate cardiac clinical forms of Chagas disease.

## Graphical abstract

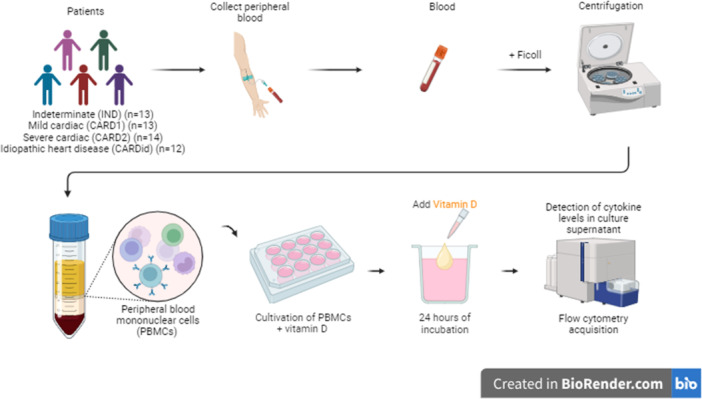

## 1 INTRODUCTION

Chagas disease or American trypanosomiasis is a chronic parasitic disease caused by the hemoflagellate protozoan *Trypanosoma cruzi*. It is a neglected tropical disease and persists as a worldwide public health problem due to the high burden of morbidity and mortality.^1^, ^2^

In a Brazilian study including 1263 patients with heart failure, Chagas disease was the cause of heart failure in 10% of the patients. Ischemic heart disease was the main cause.^3^ The pathophysiology of chronic Chagas' disease is not fully explored and clarified, presenting it with high gravity and worse prognosis than heart diseases of other etiologies. Several mechanisms have been postulated to explain the observed cardiac tissue damage better. Among these are the mechanisms of autoimmunity,^4^ parasite genetics,^5^ and host genetics^6^ which translates into the elaboration of the host‐specific immune response against *T. cruzi*. Thus, it is proposed that individuals with the chronic cardiac form, despite maintaining an effective immune response against the parasite, would not have the ability to control this inflammatory response, as individuals with the indeterminate form, and thus persisting an exacerbated inflammatory process with consequent deleterious effects to the tissue.^7^

Vitamin D is naturally synthesized in human skin through ultraviolet type B rays (UVB) or dietary sources.^8^ Active vitamin D is a fat‐soluble hormone that primarily maintains bone and calcium homeostasis, although its role in the immune response has been increasingly studied.^9^, ^10^ However, the known biological effects of vitamin D are mediated by the vitamin D receptor (VDR) and within the immune system, this receptor is found on cells such as T lymphocytes and macrophages.^11^ Vitamin D has several effects on cells of the immune system. In antigen‐presenting cells, it can induce an inhibitory effect on the expression of II major histocompatibility complex (MHC) molecules, costimulatory molecules, and other maturation‐inducing proteins (CD1a and CD83).^10^, ^12^ It promotes an increase in the chemotactic and phagocytic capacity of monocytes and cytotoxicity against tumor cells and bacteria, induction of tolerogenic dendritic cells capable of inducing T reg cells, inhibition of interleukin (IL)‐12 p70 and proinflammatory cytokines (IL‐1 and tumor necrosis factor‐α [TNF‐α]) by monocytes and macrophages.^13^, ^14^ Vitamin D interacts by participating in the regulation and differentiation of T cells by inhibiting the proliferation of T lymphocyte, the secretion of cytokines, and the progression of the cell cycle from G1a to G1b, increases the production of IL‐4, IL‐5, and IL‐10. The effect mediated by vitamin D is to inhibit IL‐12, interferon‐γ (IFN‐γ), and IL‐2, the activation of antigen‐specific T lymphocytes, and in natural killer (NK) cells.^13^ With regard to B lymphocytes, the presence of vitamin D does not contribute to the proliferation and secretion of antibodies and the production of autoantibodies, as well as blocking the differentiation of immunoglobulin‐producing B cells.^15^, ^16^ But it is worth mentioning that, at normal levels, vitamin D can regulate B cells and antibody secretion.^13^

The literature shows evidence of how vitamin D deficiency would be involved in several chronic noncommunicable diseases^17^, ^18^, ^19^, ^20^, ^21^, ^22^ and suggests it as a new risk factor for cardiovascular disease.^23^ Low serum levels of vitamin D have often been found in groups with terminal heart failure, reinforcing that this hormone plays an immunomodulatory role for these patients.^24^, ^25^ In Chagas disease, it has been shown that lower vitamin D levels suggest an association with the cardiac form.^26^ However, a large meta‐analysis described that vitamin D supplementation was not associated with reduced major adverse cardiovascular events or all‐cause mortality,^27^ and using vitamin D supplements is not recommended to reduce the risk of acute cardiovascular events.^28^ Considering the participation of vitamin D in cardiovascular health and its important function in the cells of the innate and adaptive immune response, and that immune balance in Chagas infection is an essential factor in controlling morbidity, this study aimed to evaluate the cytokine production in peripheral blood mononuclear cells (PBMC) induced by in vitro treatment with vitamin D in different chronic clinical forms of Chagas disease.

## 2 MATERIALS AND METHODS

### 2.1 Study population

Forty‐four (*n* = 44) patients with Chronic Chagas disease were selected at the Chagas Disease and Heart Failure Outpatient Clinic of the Pronto Socorro Cardiológico de Pernambuco (PROCAPE) of the Hospital Universitário Oswaldo Cruz (HUOC) of the Universidad of Pernambuco (UPE), Recife, PE. Inclusion criteria were (1) having the clinical exams for the characterization of clinical forms, such as electrocardiogram, echocardiogram, chest and esophageal X‐rays, and imaging of the colon when necessary (2) with a serological test to detect IgG reactive for Chagas infection, and (3) not reporting dysphagia and/or constipation. The selected patients were distributed into the following clinical groups: Indeterminate (IND) (*n* = 13), mild cardiac (CARD1) (*n* = 13), and severe cardiac (CARD2) (*n* = 14) and were classified by cardiologists according to the II Brazilian Consensus on Chagas disease.^1^ All individuals with Chagas disease received etiologic treatment with Benznidazole (5 mg/kg/day) between 3 and 5 years before the start of the study and were not carriers of pacemakers, infectious‐parasitic diseases, or metabolic diseases, like another recent study by our research group.^29^ Blood samples from uninfected but idiopathic heart disease (CARDid) individuals (*n* = 12) were used as study controls. Demographic and laboratory information (sex, weight, height, body mass index [BMI], total cholesterol, triglycerides, and glucose) were obtained from medical records after screening with nursing staff (Table 1). The left ventricular ejection fraction (LVEF) was obtained from the echocardiogram using the Teichholz or Simpson methods at random, depending on the examiner.

**Table 1:** Clinical and laboratory variables of the study participants.

Variables	Clinical forms				
	IND (*n* = 13)	CARD1 (*n* = 13)	CARD2 (*n* = 14)	CARDid (*n* = 12)	*p* Value
Sex					
Male	3	3	9	7	
Female	10	10	5	5	
Age (years), mean ± SD	53 ± 13.7	57.15 ± 12.83	58.71 ± 13.08	56.41 ± 10.32	0.730
Weight (kg), mean ± SD	75.23 ± 13.4	66.30 ± 9.59	69.85 ± 16.93	71 ± 13.94	0.435
Height (m), mean ± SD	1.60 ± 0.09	1.57 ± 0.10	1.61 ± 0.08	1.65 ± 0.09	0.149
BMI (kg/m^2^),^a^ median (range)	28.70 (24.09–38.67)	25.21 (21.88–32.73)	24.31 (20.9–36.21)	25.70 (19.38–37.22)	0.270
LVEF (%), (percent)	65.6 (54.0–71.9)	65.2 (55.0–71.9)	34.9 (13.4–42.0)	37.9 (20.6–68.1)	<0.0001
Cholesterol total,^a^ mean ± SD (range)	216.24 ± 36.41	199.97 ± 38.83	182.56 ± 42.30	199.22 ± 24.57	0.202
Triglycerides,^a^ median (range)	126.74 (74–285.54)	121 (63–432.2)	114 (63–178.33)	110 (60–127)	0.261
Glucose,^a^ median (range)	95.75 (85–234.2)	102 (88–140)	99 (90–117)	97.5 (79–170)	0.876

*Note*: There was variation in the N of the variables Total Cholesterol (TC), Triglycerides (TG), and Glucose (GLU) of the clinical forms. In the IND group: Total Cholesterol (*n* = 12), triglycerides (*n* = 12) and Glucose (*n* = 12). In the CARD1 group: TC (*n* = 12), TG (*n* = 12), and GLU (*n* = 11). In group CARD2: CT (*n* = 11), TG (*n* = 11), and GLU (*n* = 11), and in group CARDid: CT (*n* = 09), TG (*n* = 08), and GLU (*n* = 10). BMI: underweight (BMI < 18.5 kg/m^2^), normal (BMI 18.5‐24.9 kg/m^2^), overweight (BMI 25.0−29.9 kg/m^2^), obesity I (BMI 30.0−34.9 kg/m^2^), obesity II (BMI 35.0−39.9 kg/m^2^), and obesity III (BMI ≥ 40 kg/m^2^).

Abbreviations: BMI, body mass index; CARD1, individuals with Chagas disease in the mild cardiac form; CARD2, individuals with Chagas disease in the severe cardiac form; CARDid, individuals with idiopathic heart disease; IND, individuals with Chagas disease in the undetermined form of the disease; LVEF, left ventricular ejection fraction.

^a^ Reference values—TC: desirable: <200 mg/dL; borderline: 200–239 mg/dL; high: ≥240 mg/dL. TG: <150 mg/dL. GLU: normal: 70–99 mg/dL; decreased tolerance: 100–125 mg/dL; diabetes mellitus: >125 mg/dL.

*Source*: Update of the Brazilian Guidelines on Dyslipidemia and Prevention of Atherosclerosis,^53^ Brazilian Society of Diabetes Guidelines,^54^ and World Health Organization.^47^

### 2.2 Blood collection and serological diagnosis

Nine milliliters of peripheral blood were collected using a vacuum system (Vacutainer®) containing sodium heparin to obtain PBMC. Five milliliters of blood were collected in a dry tube to obtain serum and confirm serology for *T. cruzi* infection. Serology was performed by the Chagas Disease Reference Service of the Aggeu Magalhães Institute (IAM/Fiocruz) using two immunoenzymatic tests: recombinant Chagastest ELISA v. 4.0 and lysed Chagastest ELISA (both from Wiener lab.) according to the manufacturer's instructions. Reactive results were considered when both tests showed reactivity and nonreactive when both tests showed no reactivity, according to the II Brazilian Consensus on Chagas Disease.^1^

### 2.3 PBMC isolation and treatment with vitamin D

PBMCs were isolated by density gradient with Ficoll Hystopaque (Sigma‐Aldrich). One milliliter of PBMCs (1 × 10^6^/mL) was deposited in 48‐well plates and treated with cholecalciferol/vitamin D3 (Sigma‐Aldrich), an inactive form of vitamin D, at a concentration of 10^−7 ^M for 24 h at 37°C and 5% CO_2_, previously established through time kinetics and concentration already used by other authors.^30^, ^31^, ^32^ Furthermore, PBMCs were stimulated with phytohemagglutinin (PHA) (Cultilab) (10 µg/mL) (positive control) and without treatment (negative control). Vitamin D was dissolved in ethanol and therefore a well with 0.1% ethanol (EtOH) (Sigma‐Aldrich) was used as a control. The cells were cultured in RPMI 1640 medium containing 1% l‐glutamine 200 mM, 1% sodium pyruvate 100 mM, 0.2% sodium bicarbonate 7.5%, and 1% antibiotic (penicillin 100 IU/mL and streptomycin 100 mg/mL) (Gibco) and supplemented with 10% fetal bovine serum (Gibco). After the culture period, the plates were centrifuged (200 × *g* for 5 min at room temperature) and 300 µL of supernatant was removed and stored in microtubes at −20°C.

### 2.4 Cytokine dosage in the culture supernatant

Culture supernatants were collected for cytokine (IL‐2, IL‐4, IL‐6, IL‐10, TNF‐α, and IFN‐γ) dosage by CBA (Cytometric Bead Array; BD Biosciences), according to the manufacturer's instructions with modifications in the final reaction volume to 60 µL (20 µL of bead mix, 20 µL of sample, and 20 µL of detection reagent). The beads were acquired using the FACSCalibur flow cytometer (Beckton Dickson), located in the Nucleus for Technology Platforms (NPT)/IAM/Fiocruz, through the CellQuestPro software (Beckton Dickson) and analyzed in the FCAP Array 3.1 software (Beckton Dickson).

### 2.5 Statistical analysis

Statistical analysis of the data was performed using PRISM 8.0 Windows® software. To confirm the assumption of homogeneity of the samples, the Shapiro–Wilker test was used. For comparison between culture conditions, Student's *t* test for paired samples was used when the homogeneity assumption was confirmed, and Wilcoxon's test was used when the homogeneity assumption was not confirmed. To compare cytokine production between patient groups, we established an index (vitamin D‐treated cells/untreated cells) to normalize the data, in which, the analysis of variance test was used when the homogeneity assumption was confirmed and the Kruskal–Wallis test when homogeneity was not confirmed. For the correlation analysis between %LVEF and cytokines, Pearson's correlation test was used when the homogeneity assumption was confirmed, and Spearman's test when the homogeneity assumption was not confirmed. All statistical conclusions were made at a 5% significance level.

## 3 RESULTS

Table 1 represents the epidemiological (sex, age, weight, height, and BMI) and laboratory (glucose, triglycerides, and total cholesterol) data of the groups studied. As we can see about these parameters there was no statistically significant difference between the groups.

**Figure 1:**
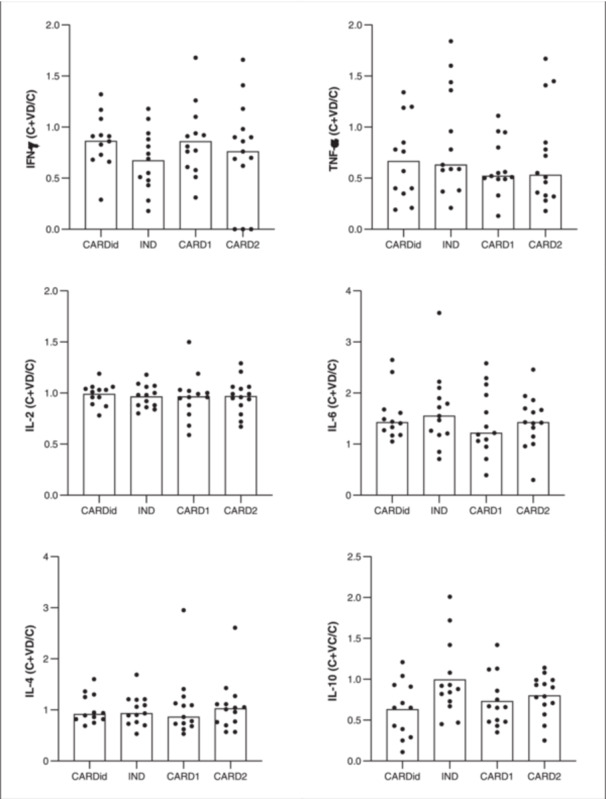
Cytokine production in PBMC culture among chronic Chagas' disease and idiopathic cardiac patients after in vitro vitamin D treatment. Horizontal bars represent the mean or median. The statistical tests used to compare cytokine concentrations between clinical forms were analysis of variance or Kruskal–Wallis. C+VD, cultures of untreated cells (C) and cultures of vitamin D‐treated cells; CARDid, individuals with idiopathic heart disease; CARD1, individuals with Chagas disease in the mild cardiac form; CARD2, individuals with Chagas disease in the severe cardiac form; IFN‐γ, interferon‐γ; IL, interleukin; IND, individuals with Chagas disease in the undetermined form of the disease; PBMC, peripheral blood mononuclear cells; TNF‐α, tumor necrosis factor‐α.

We did not find statistically significant differences between the groups when analyzing the inflammatory and anti‐inflammatory cytokine production profiles (Figure 1) in PBMC culture among patients with chronic Chagas' disease and idiopathic cardiac patients after in vitro vitamin D treatment.

**Figure 2:**
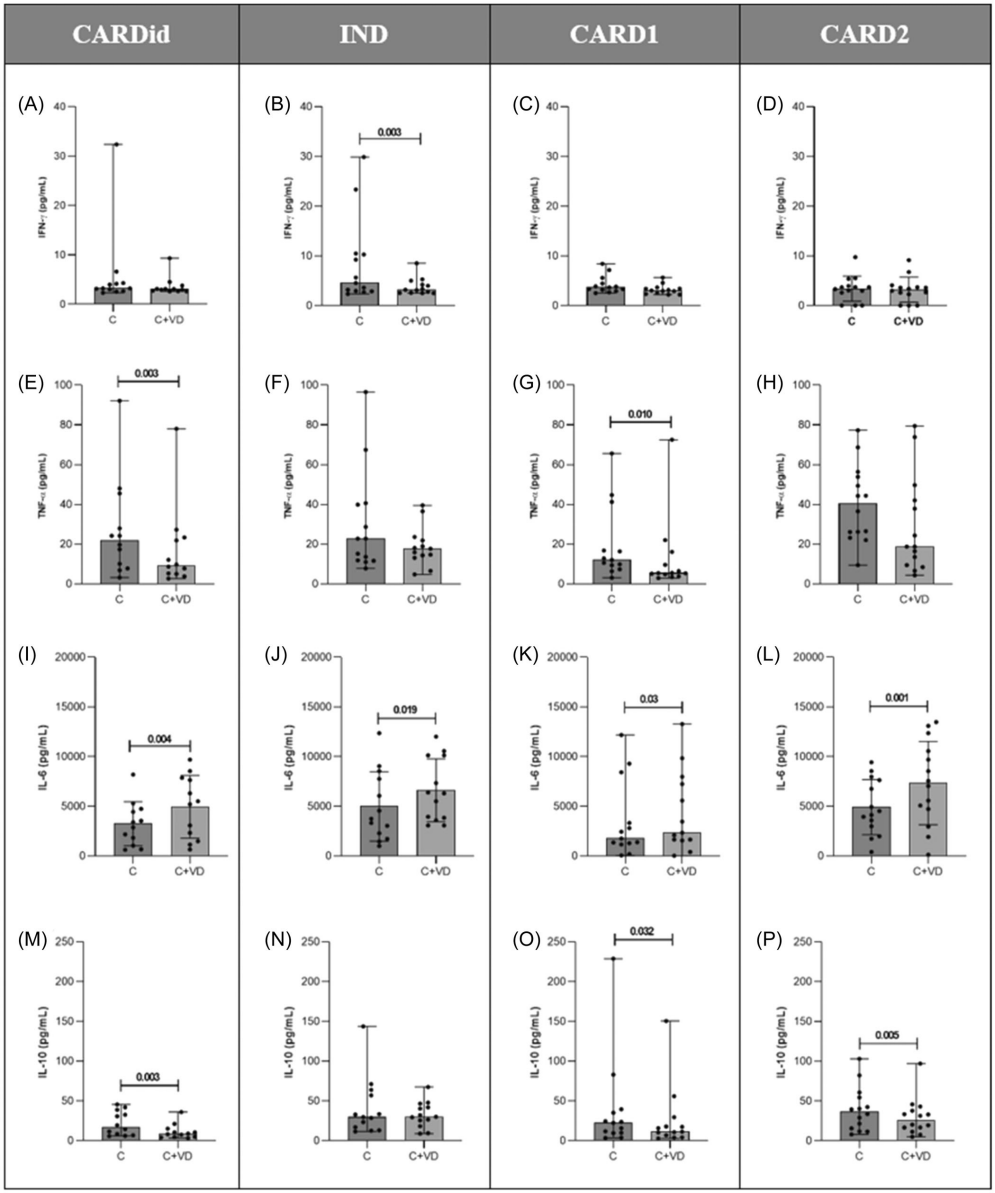
In vitro vitamin D treatment in PBMC from chronic Chagas' disease patients and idiopathic cardiac patients. CARDid (A), (E), (I), and (M)—individuals with Chagas disease with idiopathic heart disease; IND (B), (F), (J), and (N)—individuals with Chagas disease in the undetermined form of the disease; CARD1 (C), (G), (K), and (O)—individuals with Chagas disease in the cardiac form without severity and CARD2 (D), (H), (L), and (P)—individuals with Chagas disease in the cardiac form with severity. The horizontal bars represent the mean or median. The statistical tests used to compare the concentrations of cytokines between culture conditions were Student's *t* test for paired samples or Wilcoxon's test. C+VD, cultures of untreated cells (C) and cultures of vitamin D‐treated cells; CARDid, individuals with idiopathic heart disease; CARD1, individuals with Chagas disease in the mild cardiac form; CARD2, individuals with Chagas disease in the severe cardiac form; IFN‐γ, interferon‐γ; IL, interleukin; IND, individuals with Chagas disease in the undetermined form of the disease; PBMC, peripheral blood mononuclear cells; TNF‐α, tumor necrosis factor‐α.

However, when evaluating the effect of in vitro vitamin D treatment on PBMC from chronic Chagas disease carriers and idiopathic cardiac patients (Figure 2), we observed statistically significant differences between cultures of untreated cells (C) and cultures of vitamin D‐treated cells (C+VD). Vitamin D induced decreased IFN‐γ levels only in the IND group of subjects (*p* = .0034) (Figure 2B). Regarding TNF‐α, this hormone reduced the levels of this cytokine in CARDid (*p* = .0161) and CARD1 (*p* = .0105) subjects (Figure 2E,G, respectively). When analyzing IL‐6, we found a statistically significant increase in the production of this cytokine after vitamin D treatment in all patient groups, when compared to untreated cells (Figure 2I–L). Interestingly, vitamin D induced a statistically significant decrease in IL‐10 levels in all groups with cardiac manifestation (CARDid, CARD1, and CARD2). However, in IND patients, no change was observed between the culture conditions analyzed (Figure 2N). Furthermore, no statistically significant differences in IL‐2 and IL‐4 levels were observed between the groups after in vitro vitamin D treatment (results not shown).

Finally, there was no statistically significant correlation between the LVEF of Chagas disease carriers and cytokine production in cell culture treated in vitro with vitamin D (Table 2).

**Table 2:** Correlation between %LVEF and quantification of cytokines in the supernatant of PBMC culture from Chagas disease patients (IND, CARD 1, and CARD2) treated in vitro with vitamin D.

Coeficient of correlation	Cytokines					
	IFN‐γ	TNF‐α	IL‐10	IL‐6	IL‐4	IL‐2
%LVEF	0.12	0.17	−0.03	0.19	0.05	−0.11
*p* Value	0.45	0.30	0.85	0.24	0.77	0.48

*Note*: The statistical test used for correlation analysis between %LVEF and cytokines of patients with Chagas disease was Pearson's test or Spearman's test.

Abbreviations: CARD1, individuals with Chagas disease in the mild cardiac form; CARD2, individuals with Chagas disease in the severe cardiac form; IFN‐γ, interferon‐γ; IL, interleukin; IND, individuals with Chagas disease in the undetermined form of the disease; %LVEF, percent of left ventricular ejection fraction; PBMC, peripheral blood mononuclear cells; TNF‐α, tumor necrosis factor‐α.

## 4 DISCUSSION

Vitamin D is an important hormone for cellular immune responses and its serum levels are directly associated with macrophages increasing oxidative burst and inhibition of inflammatory cytokines.^33^, ^34^ Few studies have evaluated the in vitro immunomodulatory effects induced by vitamin D. Most of them have been reported in autoimmune diseases.^9^, ^35^ This is the first investigation on the effect of vitamin D on cytokine levels in the culture supernatant of peripheral blood mononuclear cells from patients with chronic Chagas disease. Thus, it is a contribution of yet another link to the understanding of the immune mechanisms that lead to the distinct manifestations of the disease.

Considering that vitamin D is intrinsically related to the mechanisms of immune response regulation, we compared the production of cytokines by cells from patients with indeterminate and cardiac clinical forms (including two different degrees of heart disease: CARD1, only patients with electrocardiographic alterations and CARD2 already with significant cardiac function impairment) through the analysis of a production index (treated cells/nontreated cells) for data normalization. In this analysis, our study did not observe statistically significant differences between the clinical forms of Chagas disease regarding the production of cytokines by PBMC treated with this hormone.

Several immunological factors have been described as important in the development of the severe clinical forms of Chagas disease. Experiments using human samples found a cytotoxic immune response among chronic symptomatic individuals, with a production of CD8+T cells and inflammatory cytokines such as TNF‐α and IFN‐γ.^36^, ^37^, ^38^, ^39^ In turn, the high frequency of CD4^+^ T lymphocytes and IL10, an anti‐inflammatory cytokine, in carriers of the indeterminate form evidence a regulatory immune environment.^36^, ^38^, ^40^, ^41^

In general, in innate immunity, vitamin D modulates the differentiation and functions of antigen‐presenting cells by inducing a decrease in costimulatory molecules and MHC‐II and consequently a decrease in the interaction of these cells with T lymphocytes. This is reflected in the decreased production of IL‐12 and IFN‐γ and the increased production of IL‐10.^42^ In T cells, vitamin D suppresses T cell proliferation and modulates cytokine production and differentiation with various effects on different T lymphocyte subtypes.^43^ Vitamin D promotes the change of Th1 (IL‐2, IFN‐γ, and TNF‐α) and Th17 (IL‐17 and IL‐21) profiles to Th2 (IL‐4, IL‐5, IL‐9, and IL‐13). The inactive form of vitamin D has been evaluated in PBMC from patients with autoimmune disease, suggesting regulatory effects in these patients.^44^ In other studies, cholecalciferol reduced the susceptibility of TCD4+ cells to HIV‐1 infection in vitro.^30^, ^31^

When we evaluated the immunomodulatory effect of vitamin D within each group studied by comparing the culture conditions of vitamin D treated and untreated cells, we demonstrated that vitamin D treatment in vitro altered the cytokine production profile, highlighting the importance of the IFN‐γ, TNF‐α, IL‐6, and IL‐10 cytokine profiles. Interestingly, only cells from IND patients responded to vitamin D showing decreased IFN‐γ production. On the other hand, vitamin D induced decreased levels of IL‐10 in the cells of the cardiac patients, except the IND patients. However, although in the group of patients with severe Chagas disease (CARD2), no statistically significant difference was observed, vitamin D induced a decrease in TNF‐α produced by the cells of the groups of patients with Chagas disease. IL‐6 plays an important pleiotropic role in the survival of CD8+T cells, improving their cytotoxic activity when the immune system is activated. In our study, IL‐6 in contact with vitamin D may be favoring the cytotoxic activity of TCD8+ lymphocytes.^45^

Considering that the Th1/Th17 and Th2 paradigm is well established in human Chagas disease and that the effects of vitamin D are of suppression of the inflammatory response, we expected that the cells of patients with chagasic heart disease would respond more inefficiently to vitamin D treatment when compared to individuals of the indeterminate form, since it has been shown that serum 25(OH)D3 levels were significantly lower in patients of the cardiac form when compared to those of the indeterminate form, suggesting an association of hypovitaminosis with chagasic heart disease.^26^ Besides the classic functions of vitamin D for the organism (regulation of serum calcium and phosphorus levels and bone mineralization) this hormone has an important role in the regulation of the renin‐angiotensin system, which is important in cardiovascular health.^46^ A study, that followed patients with cardiac insufficiency (IC) for 12 months, demonstrated a high prevalence of vitamin D deficiency in these patients and reinforced its immunomodulatory role in IC.^25^

Moreover, we believe that factors such as metabolic comorbidities (dyslipidemia, diabetes, and obesity) could interfere with this result, leading us to analyze these laboratory data. The distribution of BMI in our study was classified according to the parameters established by the World Health Organization.^47^ It is important to show that all groups studied were diagnosed as overweight (BMI 25.0–29.9 kg/m^2^). Obesity is an important risk factor for cardiovascular disease and chagasic heart disease.^48^, ^49^, ^50^ In addition, recent studies by our research group have demonstrated the profile of immunometabolism and in vitro infection with *T. cruzi* in adipose tissue, which highlights the influence of this tissue on the secretion of cytokines and inflammatory mediators in the presence of the parasite.^51^, ^52^

Our results also showed that IND individuals had higher total cholesterol and blood glucose rates, being the only group above normal reference values, according to the Update of the Brazilian Guidelines on Dyslipidemia and Prevention of Atherosclerosis^53^ and Brazilian Society of Diabetes Guidelines,^54^ although no statistically significant difference was demonstrated with the other patient groups.

Obesity and diabetes have been associated with a decrease in vitamin D levels and the risk of heart disease, and studies have shown that patients with Chagas disease have a high prevalence of comorbidities.^55^ However, the characterization of the clinical forms used in our study was more robust with the use of echocardiogram data, being possible to stratify chagasic heart disease into mild and severe, which has already been used by our research group.^29^, ^56^

However, the results of this research also showed that all groups of patients had elevated levels of the cytokine IL‐6 (an important inflammatory mediator of the immune response) when comparing the treated and untreated PBMC cultures. As already mentioned, vitamin D induces suppression of IL‐6 production.^57^ However, other work has found that vitamin D intake caused an increase or no significant change in IL‐6 levels after supplementation in a population affected by dyslipidemia, obesity, or diabetes.^58^, ^59^ However, one study has already demonstrated the same profile of increased proinflammatory cytokine in young children who supplemented vitamin D for 12 weeks to strengthen immune function.^60^ Therefore, more robust studies are needed to provide a better understanding of the association of IL‐6 with vitamin D supplementation.

The heart fraction, the ratio of ejected systolic volume to end‐diastolic volume, can be assessed by measuring LVEF.^61^ LVEF percentage (%LVEF) is determined via echocardiography. Notably, low values of this metric have been associated with a poor prognosis for heart failure or heart disease, particularly in cases of Chagasic heart disease. The limit for a normal LVEF recommended by international guidelines is less than 52% for men and less than 54% for women. The cutoff below 40% is for moderate LV systolic dysfunction and below 30% is for a severely depressed LV systolic function.^62^ However, our findings revealed no statistically significant correlation between cytokine production (Th1/Th2) and % LVEF, as presented in Table 2.

The variability of studies reinforces the premise that vitamin D is an important immunomodulator and its deficiency is closely associated with a favoring of cardiovascular diseases.^63^, ^64^, ^65^ Thus, we believe that vitamin D supplementation in Chagas infection should be investigated further and that clinical trials with vitamin D supplementation in Chagas disease should be encouraged as they are in autoimmune diseases.^13^, ^66^

Finally, it has been shown that vitamin D deficiency and consequently decreased activation of the VDR could play an important role in cardiovascular disease since it would affect cardiac contractility, vascular tone, maturation, and collagen content of cardiac tissue, as the hearts of VDR knockout mice show high hypertrophy and profound changes in heart structure.^67^ Other studies with VDR knockout mice showed stimulation of the renin–angiotensin–aldosterone system favoring hypertension, increased fluid intake, and left ventricular hypertrophy.^68^, ^69^ It is worth noting that our study used culture supernatant from mononuclear cells treated with vitamin D, and thus the detected cytokine production is the product of this cell mixture. Knowing that vitamin D needs the VDR to perform its biological functions.^10^ Studies are needed to evaluate the expression of the VDR in immune cells of patients with Chagas disease, associating it with serum vitamin D dosage.

## 5 CONCLUSION

In vitro, treatment with vitamin D revealed a difference in cytokine production by peripheral blood mononuclear cells between patients with chronic cardiac and indeterminate clinical forms of Chagas' disease, with a decrease in IL‐10 in cardiac patients. However, there were no significant changes in IL‐2 and IL‐4 levels after treatment. In addition, no significant correlation was found between LVEF and cytokine production, indicating independence of the immunomodulatory effects of vitamin D on cardiac function. These findings suggest that vitamin D may affect cytokine production differently in patients with Chagas' disease, depending on their clinical presentation. Given the possible importance of vitamin D in immune regulation and cardiovascular health, further research into vitamin D supplementation in Chagas disease is needed. In addition, studies exploring the expression of the VDR in immune cells of patients with Chagas' disease and its association with serum vitamin D levels would provide crucial information on the mechanisms underlying the effects of vitamin D in this context.

## 6 LIMITATIONS

Despite the limitations inherent in the study, we tried to minimize them to provide a solid basis for interpreting the results. All patients in this study have a history of previous parasitological treatment. Although all of them had positive serological tests for Chagas disease, it is not possible to rule out that a group of the patients had a parasitological cure as takes years to decades until serology becomes negative. This may have influenced the patient's immune response. However, clear criteria for chronic Chagas disease cure beyond a negative serology are still missing.

As for the nonexposure of PBMCs to the *T. cruzi* antigen during treatment with vitamin D, it is essential to note that these patients had already been exposed to the parasite while infected (chronic Chagas disease patients), showing a specific cellular immune response to the disease, even in the absence of the parasite. Furthermore, by the aim of the study, we sought to understand the effects of vitamin D in isolation without the interference of other immunological stimuli, such as exposure to the *T. cruzi* parasite.

Finally, other limitations of this study were the small sample size and the fact that the participants were recruited from a single institution with no internal or external validation of the results.

## AUTHOR CONTRIBUTIONS

**Kamila Kássia dos Santos Oliveira**: Conceptualization; data curation; formal analysis; investigation; methodology; software; writing—original draft; writing—review and editing. **Diego José Lira Torres**: Formal analysis; investigation; methodology. **Michelle da Silva Barros**: Formal analysis; investigation; methodology. **Leyllane Rafael Moreira**: Formal analysis; methodology. **Claudeir Dias da Silva Junior**: Methodology. **Ana Karine de Araújo Soares**: Data curation; formal analysis; methodology. **Maria da Piedade Costa Reis de Albuquerque**: Data curation; investigation; methodology; writing—original draft. **Wilson Alves de Oliveira Junior**: Data curation. **Michelle Christiane da Silva Rabello**: Formal analysis; investigation; visualization; writing—review and editing. **Virginia Maria Barros de Lorena**: Conceptualization; data curation; formal analysis; funding acquisition; investigation; project administration; resources; supervision; writing—original draft; writing—review and editing.

## CONFLICT OF INTEREST STATEMENT

The authors declare no conflict of interest.

## ETHICS STATEMENT

All patients included signed the Free Consent Form and the approaches used in this study were approved by the Research Ethics Committee of the Institute Aggeu Magalhaes/IAM, Fiocruz Pernambuco (CAAE 20049919.5.0000.5190).
